# The effects of different doses of caffeine on maximal strength and strength‐endurance in women habituated to caffeine

**DOI:** 10.1186/s12970-021-00421-9

**Published:** 2021-03-30

**Authors:** Aleksandra Filip-Stachnik, Michal Wilk, Michal Krzysztofik, Ewelina Lulińska, James J. Tufano, Adam Zajac, Petr Stastny, Juan Del Coso

**Affiliations:** 1 Institute of Sport Sciences, The Jerzy Kukuczka Academy of Physical Education in Katowice, ul. Mikolowska 72a, 40-065 Katowice, Poland; 2 Faculty of Physical Education, Gdansk University of Physical Education and Sport, 80-336 Gdansk, Poland; 3 Faculty of Physical Education and Sport, Charles University, Prague, Czech Republic; 4 Centre for Sport Studies, Rey Juan Carlos University, Fuenlabrada, Spain

**Keywords:** Bench Press, Resistance exercise, Ergogenic substances, Time under Tension, 1RM test

## Abstract

**Purpose:**

The main goal of this study was to assess the acute effects of 3 and 6 mg of caffeine intake per kg of body mass (b.m.) on maximal strength and strength-endurance in women habituated to caffeine.

**Methods:**

Twenty-one healthy resistance-trained female students (23.0 ± 0.9 years, body mass: 59.0 ± 6.6 kg), with a daily caffeine intake of 5.8 ± 2.6 mg/kg/b.m. participated in a randomized, crossover, double-blind design. Each participant performed three experimental sessions after ingesting either a placebo (PLAC) or 3 mg/kg/b.m. (CAF-3) and 6 mg/kg/b.m. (CAF-6) of caffeine. In each experimental session, the participants underwent a 1RM test and a strength-endurance test at 50 %1RM in the bench press exercise. Maximal load was measured in the 1RM test and the time under tension, number of preformed repetitions, power output and bar velocity were registered in the strength-endurance test.

**Results:**

The one-way ANOVA showed a main effect of caffeine on 1RM bench press performance (F = 14.74; *p* < 0.01). In comparison to the PLAC (40.48 ± 9.21 kg), CAF-3 (41.68 ± 8.98 kg; *p* = 0.01) and CAF-6 (42.98 ± 8.79 kg; *p* < 0.01) increased 1RM bench press test results. There was also a significant increase in 1RM for CAF-6 when compared to CAF-3 (*p* < 0.01). There was a main effect of caffeine on time under tension during the strength-endurance test (F = 13.09; *p* < 0.01). In comparison to the PLAC (53.52 ± 11.44 s), CAF-6 (61.76 ± 15.39 s; *p* < 0.01) significantly increased the time under tension during the maximal strength-endurance test.

**Conclusion:**

An acute dose of 3-to-6 mg/kg/b.m. of caffeine improves maximum strength. However, these doses of caffeine had minimal ergogenic effect on strength-endurance performance in women habituated to caffeine.

## Introduction

The acute intake of caffeine (CAF) has been found effective in enhancing exercise performance in a wide range of resistance-based exercises based on strength-endurance [1–3], and strength-power exercise modalities [4, 5]. The ergogenic effect of CAF has been found when consumed at doses ranging from 3 to 13 mg/kg body mass (b.m.) and ingested in the form of capsules 30 to 90 min before exercise [6, 7], although the use of high doses of CAF normally increases the prevalence of caffeine-associated side-effects [8]. Interestingly, the manner of CAF administration seems to be less relevant than the dosage and timing, as ergogenic effects of CAF on resistance-based exercise has been found after consuming caffeinated energy drinks [9], gels [10], and coffee [11].

Mechanisms responsible for ergogenic effects of CAF are linked to the impact of this substance on various tissues, organs and systems of the human body [12–15]. Specifically, the hydrophobic nature of CAF permits a high capacity of distribution, while its lipophilic nature enables CAF to enter all tissues, entering intracellular water and penetrating the blood-brain barrier [16]. The effect of CAF on multiple body tissues makes it difficult to accurately determine the key mechanism of action during exercise. Nevertheless, several mechanisms, such as reduced muscle pain and perceived exertion [17], enhanced fat oxidation [18], increased muscle oxygen saturation [19] and local changes within the exercising muscle [20], have been proposed to explain caffeine’s ergogenic effects, although most of them explain the effect of CAF on submaximal exercise intensity. To date, the capacity of CAF to block the fatiguing effects of adenosine seems the most plausible explanation for the wide ergogenic effects of this supplement on exercise performance [21, 22]. Briefly, evidence in animal [13] and human models [23] supports the capacity of CAF to act as an adenosine A_1_ and A_2A_ receptor antagonist, inhibiting the brake that endogenous adenosine imposes on the ascending dopamine and arousal systems [14].

Given that sex has been recognized as an important factor of athletic and sports performance through the impact of endocrine differences [24], specific recommendations for both females and males are necessary to achieve the best possible sport results with the use of CAF. However, CAF supplementation studies have primarily focused on males or a mixed gender population and little is known about the effects of CAF on muscular performance in women [25, 26]. Specifically, a systematic review [27] has suggested that the effects of CAF during resistance exercise may be reduced in women when compared to men ingesting the same CAF dosage. In addition, some of the caffeine-induced stimulant effects are of smaller magnitude in women than in men [28]. Furthermore, two recent investigations have found that the ergogenic effect of CAF may be of similar magnitude in men and women, although these investigations were carried out under aerobic conditions lasting from approximately 6 to 60 min [29, 30]. Hence, to date, it is unknown if the ergogenic effects of CAF related to resistance exercise performance observed in male subjects [1, 2, 5, 31] apply to female athletes. Additionally, the hypothesis that the magnitude of caffeine’s ergogenic effect on resistance exercise is similar in athletes of both sexes requires verification.

Only two previous studies analyzed the ergogenic effects of CAF on maximal strength and local strength-endurance in females [4, 32]. Goldstein et al. [4] showed that the acute intake of CAF (6 mg/kg/b.m.) significantly increased bench press performance (1-repetition maximum − 1RM) with no significant enhancement in the number of repetitions performed at 60 % 1RM. Likewise Sabblah et al. [32] showed significant improvements in the results of the 1RM test in the bench press exercise after ingestion of 5 mg/kg/b.m. of CAF in both male and female subjects. However, the ingestion of CAF did not produce any effect during the 1RM squat exercise and during a strength-endurance test at 40 % 1RM in a bench press exercise protocol in women, while a tendency for increased performance in the strength-endurance test was found in male subjects. Thus, the scarcity of data makes it difficult to conclude whether acute CAF intake increases resistance exercise performance, and whether the potential ergogenic effect is of a similar magnitude found in men.

There is also a lack of information on how habituation to CAF may impact the ergogenic effect of CAF in women because most previous studies did not select female participants habituated to CAF [4, 32–34]. Habitual CAF intake modifies physiological responses to acute ingestion of this stimulant by the up-regulation of adenosine receptors [33, 35]. In animal models, the acute ingestion of CAF (10 mg/kg/b.m./day for two weeks) increased the number of binding sites for adenosine in the brain cortex [36]. Then, the chronic intake of CAF results in newly-created adenosine receptors, reducing in part the competitive blockage of CAF on adenosine receptors, ultimately reducing its ergogenic effects in a progressive manner [37]. Under this background, habituation to CAF due to chronic intake would produce a progressive reduction of ergogenic effects of CAF in those athletes consuming CAF on a regular basis, because the newly created adenosine receptors may bind to adenosine and induce fatigue. A progressive habituation to the performance benefits of CAF has also been proposed in investigations with humans by comparing the ergogenic effect of CAF in naïve/low CAF consumers vs. individuals with habitual CAF intake. However, the differences in the research protocols and thresholds to consider one participant as a habitual CAF consumer make it difficult to obtain definite conclusions. Hence, the current evidence indicates that CAF habituation can decrease its ergogenic effects, but neither the time course of tolerance nor the CAF dose necessary to create habituation are known at this time.

In men, habituation to CAF reduced the ergogenic effects of acute intake of 3-to-9 mg/kg/b.m. of CAF during the bench press exercise [5] and doses up to 11 mg/kg/b.m. may be necessary to obtain minor effects of acute CAF intake on maximal muscle strength [1, 2]. Pickering and Kiely [38] suggested that the reduction in the ergogenic effects of CAF in habitual users can be modified using doses greater than the daily habitual intake. However, a study by Wilk et al. [1, 2] showed no benefits (except in maximal strength) from acute ingestion of CAF when the doses of CAF were above their habitual intake. In another study, Wilk et al. [39] found a positive effect of CAF (3 and 6 mg/kg/b.m.) on mean power output and mean bar velocity during the bench press throw in athletes habituated to CAF, and performance enhancements were obtained even when the dose of CAF did not exceed the value of habitual consumption. By using cross-sectional designs, Sabol et al. [33] and Grgic and Mikulic [34] showed that the acute effects of CAF ingestion (from 2 to 6 mg/kg/b.m.) were not impacted by participants’ habitual CAF intake as they found positive effects of CAF on resistance exercise performance in individuals with different levels of daily CAF consumption. In line with that research, a recent study by Clark and Richardson [40] conducted on habituated to caffeine men and women demonstrated that the ergogenic effect of coffee ingestion (providing 3 mg/kg/b.m. of CAF) on 5-km cycling time trial performance was similar in individuals with low and high habitual CAF consumption. However, in this latter study, the effect of sex on CAF performance enhancement was not analyzed. It should be noted that all previous studies considering the impact of habituation to CAF [1, 2, 33, 34, 39] used samples where males composed most of the study sample. Therefore, it seems that male athletes habituated to CAF may benefit from acute CAF intake but the effect of acute CAF ingestion on resistance exercise performance in women habituated to CAF is still unknown.

Therefore the main aim of this study was to assess the acute effects of different doses of CAF (3 and 6 mg/kg/b.m.) on maximal strength (1RM) and local strength-endurance during the bench press exercise in women habituated to CAF. We hypothesized that both doses, 3 and 6 mg/kg/b.m., would enhance muscular strength but none of the investigated doses would improve local strength-endurance.

## Materials and methods

### Study participants

Twenty-one healthy and strength-trained females (Table 1) volunteered to participate in the study after completing an ethical consent form. The inclusion criteria were as follows: (a) free from neuromuscular and musculoskeletal disorders, (b) habitual daily CAF intake ≥ 3 mg/kg/b.m/day [41] (c) minimum 2 years of resistance training experience (to avoid the potential interference of the learning effect of the bench press exercise technique on the results of the investigation) and participation in resistance training at least 3 days per week for the 6-month prior to enrollment in this study. All participants trained the barbell bench press as part of their regular resistance training routines. Participants were excluded when they suffered from any pathology or injury or if they were using any medications, dietary supplements or ergogenic aids which could potentially affect the study outcomes (e.g., beta-alanine, creatine, pre-workout supplements, etc.).

**Table 1:** Participants’ characteristics

Age [years]	23.0 ± 0.9
Body mass [kg]	59.0 ± 6.6
Height [cm]	168.8 ± 4.8
Body Fat [%]	19.8 ± 3.3
Resistance training experience [years]	2.9 ± 1.0
1 RM in bench press exercise [kg]	40.0 ± 9.7
1 RM in bench press exercise: body mass ratio [%]	67.9 ± 12.4
Habitual caffeine intake [mg/kg/b.m/day; mg/day]	5.8 ± 2.6; 344.4 ± 172.3
Energy intake [kcal]	2131.2 ± 185.9
Protein [% of total energy intake]	20.4 ± 3.0
Carbohydrate [% of total energy intake]	50.1 ± 3.5
Fat [% of total energy intake]	29.5 ± 2.3

All data are presented as mean ± standard deviation; *1RM* one-repetition maximum

### Habitual caffeine intake assessment

Habitual CAF intake was assessed by an adapted version of the Food Frequency Questionnaire (FFQ) proposed by Bühler et al., [42]. Household measures were employed to individually assess the amount of food consumed during the day and was obtained for the four weeks before the start of the experiment, following previous recommendations [41]. Nutritional tables were used for database construction and an experienced nutritionist calculated the daily CAF intake for each participant.

### Experimental design

This study used a randomized, double-blind, crossover design where each participant acted as her own control. In a pre-experimental session, the participants performed a familiarization protocol that included the evaluation of 1RM bench press performance and one set of the bench press performed to failure with a load 50 %1RM. Then, participants underwent three identical experimental sessions with a one-week interval between sessions to allow for complete recovery [43]. The only difference in these experimental sessions was the substance ingested which was either a placebo (PLAC; all-purpose flour), 3 mg/kg/b.m. of CAF (CAF-3; Caffeine®, Olimp Laboratories, Debica, Poland), or 6 mg/kg/b.m. of CAF (CAF-6). We selected these dosages of CAF because 3 mg/kg/b.m. represents the minimum dose that affects muscle performance during resistance-based exercise [9] while 6 mg/kg/b.m. represents a large dose that shows ergogenic effects of CAF on 1RM bench press performance in women [4]. In all trials, the substances were in opaque capsules that were ingested 60 min before the onset of testing to allow for CAF absorption as peak plasma CAF concentration is obtained 15 and 120 min after oral ingestion [44]. The blinding and randomization procedures were conducted by a member of the research team that was not directly involved in data collection. In each experimental session, the participants performed a 1RM strength test [45, 46] and a strength-endurance test using the bench press exercise [47]. During each test, power output, bar velocity, number of performed repetitions as well as time under tension were measured. The participants were instructed to maintain their usual resistance training routines, and hydration and dietary habits during the study period, including habitual CAF intake. Additionally, participants were encouraged to maintain their habitual bedtime/wake-up schedule, and they were also asked to refrain from strenuous exercise 24 h before testing and to refrain CAF intake 12 h before each trial. To replicate these standardizations, participants were requested to complete a 24-h habit record on the day before the first trial and to follow the same pattern of habits before the second and third trials. To control the diet, participants registered their food and drink intake using “MyFitnessPal” software [48] for 24 h before the testing procedures. There were no significant differences in caffeine consumption (*p* = 0.567), average calorie intake (*p* = 0.464) nor in the proportions of protein (*p* = 0.848), carbohydrate (*p* = 0.991) or fat (*p* = 0.979) intake among all three experimental trials. All testing was performed at the Strength and Power Laboratory of the Academy of Physical Education in Katowice, Poland.

### Familiarization session and one repetition maximum test

One week before the main experiment, the participants performed a familiarization session. During the familiarization session, the preliminary 1RM test and the strength-endurance test were performed. The participants arrived at the laboratory at the same time of the day as in the upcoming experimental sessions. Upon arrival, the participants cycled on an ergometer for 10 min at an intensity that resulted in a heart rate of 120–140 bpm, followed by a general upper body warm-up. Next, the participants performed 10, 5 and 3 repetitions of the bench press exercise using loads between 20 and 50 % of their estimated 1RM. Afterwards, the female participants executed single repetitions of the bench press exercise with volitional tempo of movement and 5-min rest intervals between successful trials. The load for each subsequent attempt was increased by 2.5 to 5 kg, and the process was repeated until failure. After a 5 min rest interval, the preliminary strength-endurance test was performed with a load of 50 %1RM. The strength-endurance test was terminated when momentary concentric failure occurred. No bench press suits, weightlifting belts or other supportive garments were permitted.

### Experimental protocol

Three testing sessions were used for the experimental trials and the protocols were identical except for the PLAC or CAF ingestion. All testing took place between 9:00 and 11:00 am to avoid the effect of circadian variation on the results of the investigation. The general warm-up for the experimental sessions was identical to the one used for the familiarization session. After warming-up, the participants performed the 1RM bench press test to assess upper-body maximal muscle strength. For the 1RM test, the first warm-up set included 6 to 8 repetitions with 50 % of the 1RM determined during the familiarization session. The second and third sets included 4 and 3 repetitions with 70 and 80 % of the previously measured 1RM. Participants then completed one repetition with 95 % of the previously measured 1RM. Based on whether the participant successfully lifted the load or not, the weight was increased or decreased by 2.5 kg in subsequent attempts until the 1RM for a particular session was obtained. The 1RM was defined as the highest load completed without any help of the spotters [45, 46]. Five-minute rest intervals were allowed between the 1RM attempts, and all 1RM values were obtained within five attempts.

After a 5-min rest interval, the participants completed repetitions to momentary muscular failure with a load equivalent to 50 % of the participants’ 1RM, as measured previously in the maximal muscle strength test. The use of the 1RM value measured in the previous test allowed to isolate the effect of CAF on maximal strength and strength-endurance. The end of the strength-endurance test was assumed when momentary concentric failure occurred. The concentric and eccentric phase of each repetition was performed at maximal possible velocity, but without bouncing the barbell off the chest, without intentionally pausing at the transition between the eccentric and concentric phases [45–47].

### Data acquisition

During the maximal strength test, only the load that represented the participants 1RM was recorded. During the strength-endurance test, a linear position transducer system (Tendo Power Analyzer, Tendo Sport Machines, Trencin, Slovakia) was used to evaluate bar velocity during each repetition performed in the test. Using a set external load, the system calculates power output and velocity during the concentric phase of the movement. Previous studies have shown high reliability and validity of this linear transducer (ICC = 0.970 to 0.988 [49]). The following variables were recorded for each repetition:

- REP – number of repetitions [n].
- TUT - time under tension [s].
- PP - peak concentric power [W].
- MP - mean concentric power [W].
- PV - peak concentric velocity [m/s].
- MV - mean concentric velocity [m/s]..

The peak value of power output and bar velocity was obtained from the repetition with the highest value of power output/velocity performed during the strength-endurance test. The mean value of power output and bar velocity was obtained as the average of all repetitions performed during the strength-endurance test. During the experimental sessions, a certified operator recorded all sessions by means of a camera. Time under tension and the number of performed repetitions was obtained manually from the recorded data.

### Statistical analysis

The Shapiro-Wilk, Levene and Mauchly´s tests were used in order to verify the normality, homogeneity and sphericity of the sample data. Verification of differences between the PLAC vs. CAF-3 and CAF-6 groups was performed using one-way ANOVA for repeated measures. In the event of a significant main effect, post-hoc comparisons were conducted using the Tukey’s test. Percent relative effects and the 95 % confidence intervals were also calculated. Effect Sizes (Cohen’s *d*) were reported where appropriate. Parametric effect sizes were defined as: large (*d* > 0.8); moderate (*d* between 0.8 and 0.5); small (*d* between 0.49 and 0.20) and trivial (*d* < 0.2). Statistical significance was set at *p* < 0.05. All statistical analyses were performed using Statistica 9.1 and were presented as means ± standard deviations.

## Results

**Fig. 1:**
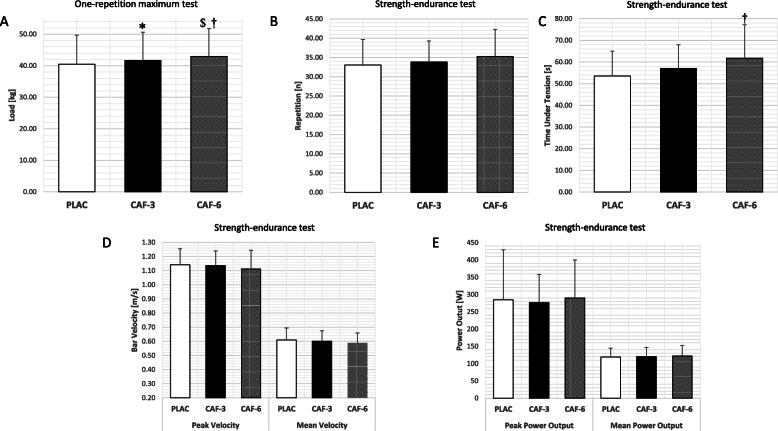
Dose–response effects of caffeine ingestion on maximal strength and strength-endurance. **a** Load in the one-repetition maximum test; **b** Number of repetitions in the strength endurance-test; **c** Time Under Tension in the strength-endurance test; **d** Peak and Mean bar velocity in the strength endurance-test; **e** Peak and mean power output in the strength endurance-test. Data are mean ± standard deviations for 21 women habituated to caffeine. *Significant difference (*p* < 0.05) between CAF-3 and PLAC. †Significant difference (*p* < 0.05) between CAF-6 and PLAC. $Significant difference (*p* < 0.05) between CAF-3 and CAF-6

The one-way ANOVA revealed a statistically significant main effect of substance for 1RM (F (2, 19) = 14.74; *p* < 0.01) and time under tension (F (2, 19) = 13.09; *p* < 0.01; Table 2; Fig. 1). The post-hoc tests revealed a significant increase in 1RM after the intake of CAF-3 (41.68 ± 8.98 kg; *p* = 0.01) and CAF-6 (42.98 ± 8.79 kg; *p* < 0.01) compared to PLAC (40.48 ± 9.21 kg), as well as a difference between CAF-6 compared to CAF-3 (*p* < 0.01; Table 3; Fig. 1). The post-hoc tests also revealed a significant increase in time under tension after the intake of CAF-6 compared to PLAC (61.76 ± 15.39 vs. 53.52 ± 11.44 s, respectively *p* < 0.01), and lack of significant changes in time under tension between CAF-3 and PLAC (57.05 ± 10.9 s *p* = 0.20). There were no significant main effects of substance for the number of performed repetitions (*p* = 0.18), mean power output (*p* = 0.56), mean bar velocity (*p* = 0.45), peak power output (*p* = 0.75) and peak bar velocity (*p* = 0.23; Table 2) during the strength-endurance test.

**Table 2:** Summary of performance data in bench press exercise after the ingestion of a placebo (PLAC) or 3 (CAF-3) or 6 mg/kg/b.m. of caffeine (CAF-6) in women habituated to caffeine

Variable	PLAC (95 % CI)	CAF-3 (95 % CI)	CAF-6 (95 % CI)	*p*
1RM [kg]	40.48 ± 9.21 (36.29 to 44.67)	41.68 ± 8.98 (37.59 to 45.76)	42.98 ± 8.79 (38.98 to 46.98)	< 0.01
REP [n]	33.05 ± 6.59 (30.05 to 36.05)	33.81 ± 5.46 (31.32 to 36.30)	35.29 ± 6.99 (32.10 to 38.47)	0.18
TUT [s]	53.52 ± 11.44 (48.3 to 58.7)	57.05 ± 10.90 (52.1 to 62.0)	61.76 ± 15.39 (54.8 to 68.8)	< 0.01
MP [W]	119 ± 25 (107 to 130)	120 ± 27 (107 to 132)	122 ± 31 (108 to 137)	0.56
PP [W]	284 ± 145 (219 to 350)	277 ± 82 (239 to 314)	290 ± 110 (240 to 340)	0.75
MV [m/s]	0.61 ± 0.08 (0.58 to 0.65)	0.60 ± 0.07 (0.56 to 0.63)	0.59 ± 0.07 (0.56 to 0.63)	0.45
PV [m/s]	1.14 ± 0.11 (1.09 to 1.19)	1.14 ± 0.10 (1.09 to 1.18)	1.11 ± 0.13 (1.05 to 1.17)	0.23

All data are presented as mean ± standard deviation; *CI* confidence interval; *1RM* one-repetition maximum; *REP* number of performed repetitions; *TUT* time under tension; *MP* mean power output; *PP* peak power output; *MV* mean bar velocity; *PV* peak bar velocity

**Table 3:** Pairwise differences in bench press performance after the ingestion of a placebo (PLAC) or 3 (CAF-3) or 6 mg/kg/b.m. of caffeine (CAF-6) in women habituated to caffeine

Variable	Comparison	*p*	Effect size (Cohen’s *d*)	Relative effect [%]
1RM [kg]	PLAC vs. CAF-3	0.01	0.11 – trivial	3.5
	PLAC vs. CAF-6	< 0.01	0.28 – small	6.9
	CAF-3 vs. CAF-6	< 0.01	0.14 – trivial	3.2
REP [n]	PLAC vs. CAF-3	0.80	0.13 – trivial	4.4
	PLAC vs. CAF-6	0.17	0.33 – small	9.5
	CAF-3 vs. CAF-6	0.45	0.24 – small	6.2
TUT [s]	PLAC vs. CAF-3	0.20	0.32 – small	8.6
	PLAC vs. CAF-6	< 0.01	0.61 – moderate	17.6
	CAF-3 vs. CAF-6	0.06	0.35 - small	8.7
MP [W]	PLAC vs. CAF-3	0.94	0.04 – trivial	0.8
	PLAC vs. CAF-6	0.36	0.11 – trivial	2.4
	CAF-3 vs. CAF-6	0.56	0.07 – trivial	1.6
PP [W]	PLAC vs. CAF-3	0.84	0.06 – trivial	1.8
	PLAC vs. CAF-6	0.93	0.05 – trivial	5.1
	CAF-3 vs. CAF-6	0.63	0.13 – trivial	3.9
MV [m/s]	PLAC vs. CAF-3	0.44	0.13 – trivial	-2.2
	PLAC vs. CAF-6	0.29	0.26 – small	-2.7
	CAF-3 vs. CAF-6	0.96	0.14 – trivial	0.1
PV [m/s]	PLAC vs. CAF-3	0.98	0.00	0.1
	PLAC vs. CAF-6	0.47	0.25 – small	-1.7
	CAF-3 vs. CAF-6	0.60	0.26 small	-1.5

All data are presented as mean ± standard deviation; *1RM* one-repetition maximum; *REP* number of performed repetitions; *TUT* time under tension; *MP* mean power output; *PP* peak power output; *MV* mean bar velocity; *PV* peak bar velocity

## Discussion

Due to the lack of data regarding this topic, the aim of the current investigation was to assess the acute effects of 3 and 6 mg/kg/b.m. of CAF on maximal strength and strength-endurance during the bench press exercise in women habituated to CAF. The main finding of the study was that, compared to the ingestion of PLAC, the acute intake of CAF-3 and CAF-6 provided an ergogenic effect on the 1RM bench press performance. There was also a significant increase in 1RM values when comparing CAF-6 to CAF-3 (*p* < 0.01), suggesting a dose-response effect of caffeine on maximal strength in women habituated to caffeine. Furthermore, CAF-6 increased the TUT during the bench press with 50 % 1RM performed to failure. However, there were no significant differences in REP, MV, PP, MP, or PP among the conditions. Thus, acute CAF ingestion of 3 to 6 mg/kg/b.m. may be useful for acutely improving maximum muscle force production in habituated resistance-trained women, but likely would not have an effect on their muscular strength-endurance.

The increases in 1RM after CAF intake in the present study are consistent with previous studies on females [4, 32] and males [1, 2]. Both doses, CAF-3 and CAF-6, were effective in enhancing maximal strength during the bench press while the magnitude of the effect was trivial-small in both cases. While these effects on maximal muscle strength may be considered as minor in statistical terms, the magnitude of the benefit (3.0 to 6.2 % for CAF-3 and CAF-6, respectively) may be significant in competitions where victory is obtained by a margin of lower magnitude that the one found in this study [50]. Previous studies assessing the effects of CAF on 1RM performance in female subjects were carried out in non-homogeneous groups in terms of daily CAF intake by participants [4, 32]. In the study of Goldstein et al. [4], CAF consumption within subjects ranged from 0 to 416 mg per day and in that of Sabblah et al. [32], daily CAF intake was not reported. In the current investigation, the self-reported daily ingestion of CAF amounted to 5.8 ± 2.6 mg/kg/b.m./day (147 to 783 mg/day). In this case, although the group was also heterogeneous in terms of daily CAF ingestion, this is the first investigation to use a sample of women habituated to CAF, with at least 3 mg/kg/b.m. per day. Nevertheless, it is still possible that the difference in the level of habituation to caffeine in the participants of the current study played a role in the between-individual response variation to acute caffeine intake [51]. According to Svenningsson et al. [52] and Fredholm et al. [53], habitual CAF intake may modify physiological responses to acute CAF ingestion by the up-regulation of adenosine receptors. Furthermore, constant exposure to CAF could impact CAF metabolism by inducing an accelerated conversion of CAF into dimethylxanthines by the cytochrome P450. Therefore, progressive habituation to the performance benefits of CAF intake has been recognized in humans when it is consumed chronically [37, 54]. However, Pickering et al. [55] suggested that the reduction in the ergogenic effects of CAF in habitual users can be modified using doses greater than the daily habitual intake. In the current investigation, the acute CAF doses (especially CAF-3) did not exceed the value of habitual consumption. Interestingly, we observed a dose-response effect of caffeine on 1RM (Fig. 1 a) and a similar dose-response effect on REP and TUT (Fig. 1b c). These outcomes indicate that the magnitude of the ergogenic effect of caffeine was higher with CAF-6 than with CAF-3 in several bench press performance variables. This was probably associated with the level of habituation to caffeine in our study sample (i.e., 5.8 ± 2.6 mg/kg/b.m.), as the dose of CAF-6 was the only dose close to their daily caffeine consumption. Therefore, in women habituated to caffeine, the use of acute caffeine ingestion close to their daily intake may produce greater ergogenic benefits than lower doses of caffeine. Nevertheless, although the current investigation found a positive effect of CAF on 1RM bench press results in females habituated to CAF, it is still possible that the effect of this substance is higher in unhabituated individuals. To the best of our knowledge, only one previous study analyzed 1RM changes of the upper limbs in a group of habitual users but included male subjects [1, 2]. The study of Wilk et al. [1, 2] showed an increase in 1RM bench press performance after the intake of 9 and 11 mg/kg/b.m. of CAF compared to PLAC which is consistent with our results. Interestingly, the same ergogenic effect was found in the current investigation in a sample of female-only participants by using lower CAF dosage.

Despite the fact that, compared to the ingestion of PLAC, the acute intake of CAF-3 and CAF-6 provided an ergogenic effect on the bench press 1RM performance we did not observe such an effect on the number of performed REP during the BP performed to muscular failure. This result is compatible with the study on female [4, 32] as well as male subjects [1, 2, 31, 34]. These studies did not show a significant impact of acute CAF intake on the number of performed REP, regardless of the level of habitual CAF consumption and used CAF dose. Therefore, it can be suggested that similar to the effect of CAF intake on maximal strength, the level of CAF habituation has no effect on the number of performed REP following acute CAF intake. However, this conclusion can only be related to women, because according to Sabblah et al. [32] there is a tendency for a lower effect of CAF on strength-endurance performance in women compared to men which requires further research.

Despite the fact that our study did not show significant changes in the number of performed REP between CAF-3 and CAF-6 compared to PLAC, a significant increase was registered in TUT after the intake of CAF-6 compared to PLAC. Such changes were not observed after the ingestion of the lower dose of CAF (3 mg/kg/b.m.). Most studies that have analyzed the effect of CAF intake on exercise volume have demonstrated an ergogenic effect of CAF on this variable evaluated by either using the number of performed REP or tonnage. Only one previous study analyzed the impact of CAF intake on TUT by using a cross-sectional experiment with a group that ingested 5 mg/kg/b.m. of CAF or PLAC before performing the strength-endurance bench press exercise at 70 % 1RM performed to muscular failure [7]. This investigation showed a significant decrease in TUT in the group that received CAF compared to the control group, what is contradictory to the results of our study. Conflicting results between the presented studies and those of Wilk et al. [7] can be related to the gender difference of the subjects (male vs. female). Sex may have a significant effect on skeletal muscle morphology and function [35], muscle substrate utilization and neuromuscular activation [56]. Women commonly have a higher proportion of type I fibers, greater muscle capillary density [57] with distinct glycolytic and oxidative capacities [58, 59]. These sex differences may be highly beneficial for endurance exercise powered by slow oxidative metabolism [60]. Furthermore, type I fibers contract with greater tension in response to increased concentration of CAF than type II fibers, and it has been previously suggested that the ergogenic benefit of CAF may be more pronounced in slow twitch muscles [61–63]. This can partially explain the equivocal nature of previous findings on the ergogenic effects of CAF and can also explain increased TUT in the group of females compared to the decrease of TUT observed for men. Yet, this explanation remains speculative until further investigation confirms an greater effect of CAF in women vs. men in local strength-endurance tests because current evidence shows similar ergogenic benefits in cycling endurance activities [29, 30], and a tendency for a lower effect of CAF on strength-endurance performance in female subjects [32]. However, for assessing local strength-endurance and to test the effectiveness of dietary supplements during resistance exercise, the use of crossover designs and the evaluation of not only the number of REP, but also TUT may be recommended [7].

The experimental design employed in this investigation presents some limitations that should be addressed to enhance the application of the study outcomes. First, we used a convenience sample of twenty-one healthy and strength-trained females to determine the effect of acute caffeine intake on maximal strength and strength-endurance in women habituated to caffeine. Although the results of this study showed an ergogenic effect of caffeine of small magnitude to enhance 1RM bench press performance, the high inter-individual variability in the values of 1RM suggests the need of confirming the results of the current experiment in future studies with higher sample sizes or by using a more homogeneous population, with similar values of maximal strength. Second, as the study sample was composed of individuals with a moderate level of resistance exercise experience, the translation of the research outcomes to highly trained women in resistance exercise should be made with caution. Third, the participants in this study were selected because they were at least moderate caffeine consumers. It is still possible that the ergogenic effect of caffeine in regards to maximal strength and strength-endurance can be greater/smaller in women with different levels of habituation to caffeine. Last of all, this study did not include blood samples and thus we have no data to determine whether participants performed the testing with peak serum caffeine concentration. In addition, we cannot confirm if the ingestion of 6 mg/kg/b.m. of CAF produced higher serum caffeine concentrations than 3 mg/kg/b.m. of CAF. More research is needed to determine the best dosing of pre-exercise caffeine supplementation to improve resistance exercise performance in women depending on their training level and experience, habituation of caffeine and type of strength exercise used.

## Conclusions

An acute dose of CAF between 3 and 6 mg/kg ingested 60 min before resistance exercise increased 1RM strength and TUT during the bench press exercise performed to concentric failure with 50 % 1RM in females habituated to CAF. In contrast, no significant changes were observed in the number of performed REP, power output and bar velocity during the strength-endurance test at 50 % of 1RM. We observed a dose-response effect of caffeine on 1RM and a trend for a dose-response effect on REP and TUT, suggesting that the use of a dose of caffeine close to individuals’ daily caffeine consumption may be recommended in athletes habituated to caffeine. This is a novel finding as it is the first investigation to examine CAF supplementation among young, trained females habituated to CAF consumption. From a practical perspective, the use of CAF may be recommended to increase muscle strength in women habituated to CAF, but dishabituation to this substance may be necessary to obtain a wider ergogenic effect of CAF in strength-endurance athletes. Although the results of the present study are associated to resistance exercise, the ergogenic benefit of acute caffeine intake may be translated to other sports requiring high levels of muscular strength in sport-specific situations (e.g., rugby, combat sports, etc.). Considering that a large part of resistance training includes exercises based on strength-endurance, the daily use of CAF may need to be avoided to reduce habituation to the ergogenic effect of CAF on this capacity. In addition, dishabituation to CAF may be useful to lessen the drawbacks associated to chronic ingestion of CAF, such as excessive nervousness insomnia and diuresis [19]. In this regard, supplementation with CAF should only be recommended for training days with very high exercise intensity or before competition. The results of our study and its application refer only to the upper limbs and thus, they should not be translated to other forms, volumes, or intensities of resistance exercise.

## Data Availability

The datasets used and/or analyzed during the current study are available from the corresponding author on reasonable request.
